# Advancement of an Infra-Red Technique for Whole-Field Concentration Measurements in Fluidized Beds

**DOI:** 10.3390/s16030300

**Published:** 2016-02-27

**Authors:** Jose A. Medrano, Niek C. A. de Nooijer, Fausto Gallucci, Martin van Sint Annaland

**Affiliations:** Chemical Process Intensification, Department of Chemical Engineering and Chemistry, Eindhoven University of Technology, De Rondom 70, 5612 AP Eindhoven, The Netherlands; j.a.medrano.jimenez@tue.nl (J.A.M.); n.c.a.d.nooijer@tue.nl (N.C.A.d.N.); f.gallucci@tue.nl (F.G.)

**Keywords:** IR, fluidized bed membrane reactor, mass transfer, concentration polarization

## Abstract

For a better understanding and description of the mass transport phenomena in dense multiphase gas-solids systems such as fluidized bed reactors, detailed and quantitative experimental data on the concentration profiles is required, which demands advanced non-invasive concentration monitoring techniques with a high spatial and temporal resolution. A novel technique based on the selective detection of a gas component in a gas mixture using infra-red properties has been further developed. The first stage development was carried out using a very small sapphire reactor and CO_2_ as tracer gas. Although the measuring principle was demonstrated, the real application was hindered by the small reactor dimensions related to the high costs and difficult handling of large sapphire plates. In this study, a new system has been developed, that allows working at much larger scales and yet with higher resolution. In the new system, propane is used as tracer gas and quartz as reactor material. In this study, a thorough optimization and calibration of the technique is presented which is subsequently applied for whole-field measurements with high temporal resolution. The developed technique allows the use of a relatively inexpensive configuration for the measurement of detailed concentration fields and can be applied to a large variety of important chemical engineering topics.

## 1. Introduction

Good mixing, high heat and mass transfer rates, thermal homogeneity and efficient contact between the fluid and solids phases make the use of fluidized beds advantageous over other reactor types for many different applications. Fluidization can be described as the operation where a packed bed of solid particles is transformed into a fluid-like state when particles are suspended in a gas or liquid. When the pressure gradient is enough to support the bed weight per unit area, the particles start to behave in many aspects like a liquid. At such conditions, the bed is at minimum fluidization conditions (*u_mf_*). Once the gas velocity exceeds *u_mf_*, gas bubbles are formed and rise through the bed carrying along with them the particulate phase, thus providing a good solids mixing in the bed and good properties in terms of heat and mass transfer rates [1]. However, fluidized beds do not only show advantages over other reactor systems but they also have some undesirable characteristics, as stated by Kunii and Levenspiel [2], such as the non-uniform residence time for the solids phase in circulating beds, attrition and elutriation of the particles or agglomeration and sintering of particles, which might strongly affect fluidized bed characteristics. Furthermore, the scaling-up of a fluidized bed is not as straightforward as for other systems because the system dimensions modify the fluidization characteristics and models developed to describe the behavior of these reactors are very complex and still lack predictive capabilities.

The use of fluidized beds has also been extended to physical operations such as heat exchangers, drying of solids, coating of metals and adsorption. In the last few years also, novel reactor concepts such as chemical looping [3,4,5] and membrane reactors [6,7] have been proposed in the literature. Both reactor concepts have in common that the efficiencies of the processes are improved by integrating CO_2_ capture, which after sequestration can largely contribute to the required reduction in anthropogenic greenhouse gas emissions. Fundamental knowledge of these novel systems has lately gained attention in order to promote both technologies to a more mature state as described in recent works in the literature [8,9,10]. Extensive information on these technologies can be found in the latest reviews [11,12,13,14].

The development of phenomenological models for a proper design of large-scale fluidized bed reactors is of special interest for the scientific community as well as for industry. Different phenomenological models for fluidized beds have been developed and they can be classified into three different levels depending on the sophistication, complexity and the main assumptions and correlations used [15]. An overview of these levels is depicted in Table 1.

All phenomenological models are described by two main characteristics that are strongly related: hydrodynamics and mass transfer rates. Hydrodynamics of a fluidized bed determine bubble properties and also include the description of different phases in the bed, such as the wake and cloud phases of a bubble, solids circulation and bed porosity. On the other side, mass transfer has an enormous impact on the performance of fluidized beds and is related to the gas exchange between the gas inside bubbles (named bubble phase) and the gas inside the solids phase (referred to as the emulsion phase). In solid catalyzed gas-phase reactions or in physical processes such as drying, the extent of mass exchange determines the overall performance of the process.

The hydrodynamics of fluidized beds have been studied by many researchers using different experimental techniques like X-ray [19], Particle Image Velocimetry (PIV) [20], Digital Image Analysis (DIA) [9], Magnetic Resonance Imaging (MRI) [21], Positron Emission Particle Tracking (PEPT) [22], Computed Automated Radioactive Particle tracking (CARPT) [23], Electrical Capacitance Tomography (ECT) [24], Magnetic Particle Tracking (MPT) [25], electrostatic probes [26], or pressure sensors [27]. All show some advantages and disadvantages that are related to properties such as temporal and spatial resolution, limitations to pseudo-2D reactors instead of 3D systems, or invasive/non-invasive techniques. Thanks to all these techniques, many correlations are available in the literature and the hydrodynamics can be reasonably well described for many cases.

On the contrary, the mass exchange between the bubble and emulsion phases is still not well described in the literature. Early-state descriptions of the mass exchange phenomena which were done by Davidson and Harrison [1] and Rowe [28] are still accepted for the explanation of the gas exchange. The interphase mass transfer in fluidized beds is generally considered to proceed via two different mechanisms that are usually assumed additive: convection via through-flow and diffusion. All models describing the mass exchange between the two phases assume a uniform concentration inside the bubbles. However, this assumption should be carefully reconsidered, as pointed out by Patil [29] and Dang [30]. The fact that a uniform concentration is suggested inside the bubble is also related to the difficulties in obtaining proper whole-field measurements of the gas concentration in fluidized beds. Experimental measurements of mass transfer rates between the bubble and emulsion phases have normally been carried out using invasive techniques consisting of local measurements of a gas tracer injected into an incipient fluidized bed [31]. In this case, concentrations can be measured in a single point inside the bubble phase as well as in the emulsion phase. Moreover, the placement of immersed elements in the bed affects the properties of the fluidized suspension and the behavior of an injected bubble might deviate from the behavior of a bubble in a freely bubbling bed and may not be well representative. Therefore, it is important to develop novel non-invasive techniques for the measurement of mass transfer rates in fluidized beds, and, in particular, techniques that do not suffer from critical disadvantages such as high investment costs or the use of gases with high toxicity, such as optical techniques based on X-ray, MRI or colored gases like NO_2_ [28].

**Figure 1:**
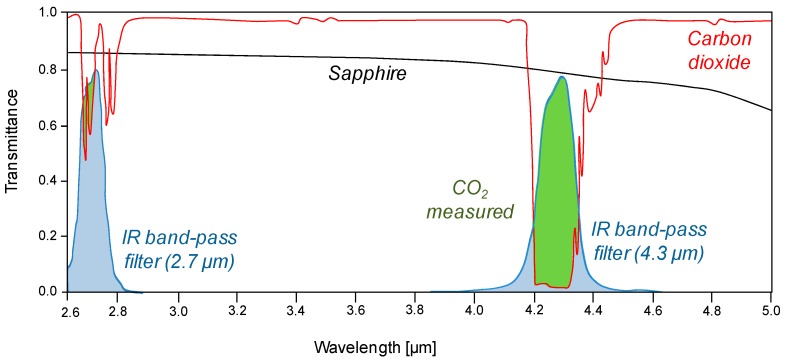
Schematic representation of the IR absorption when using CO_2_ as tracer gas in a sapphire column.

The use of IR properties of the materials has recently been applied successfully by Dang *et al.* for a first stage development of a novel technique able to measure instantaneous whole-field concentration profiles with high spatial and temporal resolution, without compromising too much on the costs of the technique [30]. In this IR transmittance technique, CO_2_ was used as tracer gas, which was introduced into a pseudo-2D fluidized bed reactor made of sapphire. An external IR source (anodized heated plate) was used and the IR absorption of CO_2_ was selectively detected with an IR camera mounting a band-pass filter, which limits the detected wavelength range to the range where the CO_2_ absorption is at a maximum. A representation of the IR absorption with the technique is shown in Figure 1, where the transmittance of sapphire is depicted together with the CO_2_ transmittance spectrum and the one for the filter installed in the IR camera. From this figure, it is first observed that sapphire has a high transmittance over the entire range, thus almost all the generated IR irradiation intensity can be detected by the camera. A band pass filter at a central wavelength at 4.26 µm was installed into the camera. This filter (represented in light blue) limits the range over which the camera detects IR radiation, thus providing an increase in detection resolution. When CO_2_ is fed to the column, it absorbs IR radiation. The decrease in IR intensity in the wavelength range of the filter is measured with the camera and can be correlated to gas concentrations. The higher the overlap of the CO_2_ absorption and transmittance of the IR band-pass filter of the camera (green area in Figure 1), the higher the resolution of this technique. It can also be seen in Figure 1 that the use of a band-pass filter at a central wavelength at 2.7 µm would give a very poor resolution which is even in the range of the noise of the technique, as further explained in the next section.

The IR technique was applied to the case of a single bubble injection into a system at minimum fluidization conditions, as well as to fluidized beds operated in the turbulent regime. Although very good results with high resolution were obtained with this technique, a very small pseudo-2D column was used, where wall effects affected the results significantly, which are thus not representative of the behavior of larger fluidized beds. When a bubble was injected into the bed, it occupied almost the whole surface and collapsed after a few milliseconds, limiting bubble growth along the axial position. Moreover, the use of sapphire makes scaling-up very expensive.

The study presented in this work represents a second stage development of the IR transmittance technique. The main objective is to find a solution to the two main drawbacks faced during the first stage development: use of expensive reactor material (sapphire) and wall effects due to the small size of the reactor. In addition, a thorough investigation is carried out on the influence of all relevant variables on the performance of the technique, including its experimental accuracy. Finding a solution to these limitations makes the technique more affordable for larger-scale investigations on the phenomenon of mass transfer in fluidized bed reactors and many other applications. The paper first discusses the new system selected for the IR technique among different options. Subsequently, the calibration of the technique is presented considering all possible variables affecting the technique. The newly-developed technique has been applied to a few different cases and these results will be discussed in Section 3. Finally, the main conclusions and guidelines for using this technique will be given.

## 2. Description of the IR Transmittance Technique

### 2.1. Infrared Technique and Discussion of the Selected System

When developing a novel technique, many factors should be considered in order to make the system easy, robust and affordable for lab-scale applications. Infrared radiation is an inherent property for any material at a temperature above 0 K. Every object emits radiation in the IR wavelength and the intensity of the radiation depends on its temperature. Nowadays there are many cameras equipped with IR detectors that can measure the IR emitted, thus transforming it into digital signals. IR cameras have already been applied in many different fields, such as electrical inspections of substations, the location of radiant heating pipes and the detection of insulation leaks in refrigeration systems.

The molecular vibrational frequencies of many gases like CH_4_, CO_2_ or CO lie in the IR wavelength range and they can absorb IR radiation emitted by a heated element. When molecules absorb IR radiation, transitions occur from a ground vibration state to an excited vibrational state, which provokes a change in the dipole moment of the bonds as a result of bond expansion and contraction. This IR absorption can be detected via a camera by a decrease in the intensity received, which is a phenomenon described by Lambert-Beer’s law. Transmittance and absorbance are two terms directly correlated and that will be used in this work for the discussion of the results. They are defined as stated in Equations (1) and (2):

(1) $$T=\frac{I}{I_{0}}$$

(2) $$A=-\text{ln}\frac{I}{I_{0}}$$

where *I*_0_ is the IR radiation measured by the detector in absence of a gas absorbing IR radiation and *I* is the intensity measured once gas is placed in between the IR source and the detector. As described by Lambert-Beer’s law, this absorbance is proportional to the gas concentration as defined in Equation (3), where *C* is the gas concentration in mol/L, ℓ is the target length, *ɛ* is the molar absorbance (mol^−1^∙cm^−2^) and *a* is a function of ℓ and ɛ.

(3) A=−εℓC=aC

The measurement principle is based on the selective absorbance of a gas, which is related to its concentration in the column. In order to further develop the technique, it is important to note that the experimental setup can be divided into four different parts: IR camera, column, gas phase and IR source. While the IR camera and IR source are standard elements, the study of mass transfer in fluidized beds using IR techniques requires certain specifications for the column and the gas phase, which are discussed next.

The column used by Dang *et al*. [30] was made of sapphire, which has a high transmittance over a wide range of wavelengths in the IR spectrum. It covers the whole range of the IR detector mounted in the camera (from 1–5 μm) and makes the selection of a proper tracer gas much easier. A high transmittance over a wide range is the main desired property for the column material, but there are several other properties that should also be addressed when optimizing the experimental technique: low index of refraction, hardness, insolubility in water, low cost, clarity for VIS light and that it is rapidly available. Many different options have been considered and some of them have been refused because they do not possess all the desired properties. For instance, silicon is not clear for VIS light, even though it has the other desired properties. Sodium chloride has a high solubility in water, while sapphire is too expensive for scaling up. The material that shows the best compromise between all the desired properties is quartz. It is a hard and clear material which can be easily obtained and is not as expensive as other materials. Therefore, quartz has been selected, although it has a narrower transmittance range than sapphire, which restricts the selection for the gas phase.

CO_2_ shows an intense IR absorption peak at a wavelength of 4.26 μm, where the quartz transmittance is poor, which implies that CO_2_ cannot be used as gas phase with quartz as column material. In order to find a suitable tracer gas, the gas needs to have an intense IR absorption in the range where the column material has a high transmittance, as well as selective absorption, is rapidly available, cheap and safe. According to these characteristics, propane has been selected as the gas which gives the best compromise between the desired properties. Propane has a strong absorption peak at 3.5 μm and is much stronger than, for example, CH_4_. Both absorb IR radiation at the same wavelengths corresponding to the carbon-hydrogen bonds. However, in the case of propane the larger number of bonds increases the strength of the peak. Formaldehyde has also been considered, but due to its toxicity it has been refused as tracer gas.

**Figure 2:**
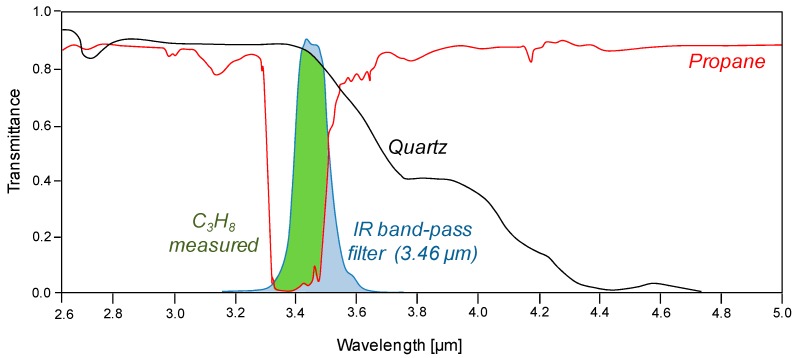
Schematic representation of the IR absorption when using C_3_H_8_ as tracer gas in a quartz column.

For the system based on a quartz column and propane as tracer gas, a band-pass filter is required in order to limit the range in the IR spectrum in which the camera detects IR radiation. A representation of the IR absorbance using this system is given in Figure 2. A more detailed description on the whole system is given in the next section.

### 2.2. Description of the Setup

**Figure 3:**
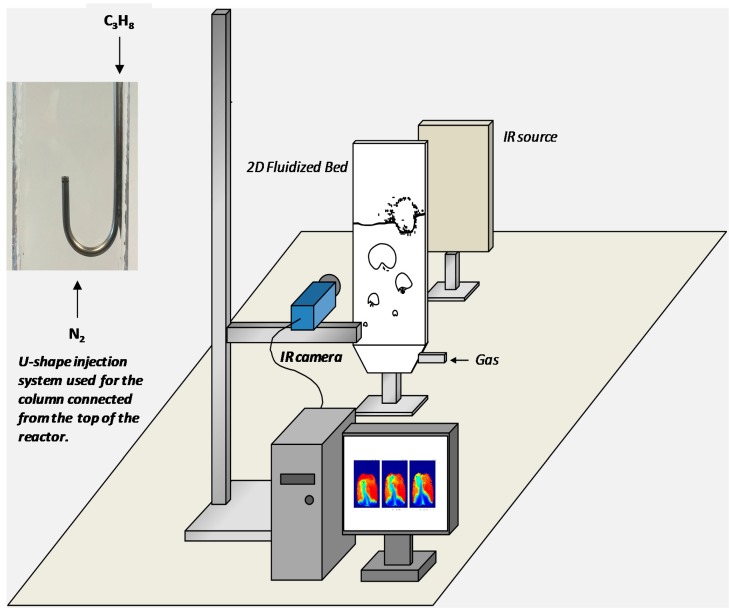
Schematic representation of the experimental setup used in this work for the calibration of all variables in the technique and a detailed image of the injection system.

The experimental setup consists of four different elements perfectly aligned in order to ensure that IR radiation is perpendicular to the detector of the camera (see Figure 3). IR radiation is generated via a heated anodized aluminum plate kept at 430 °C. The temperature of the plate (300, 150 and 20 mm in height, width and depth, respectively) is controlled using electrical tracing wires located inside the IR source. Perfectly aligned to the IR source, there is an IR camera (model SC7650 by FLIR Systems, Frankfurt, Germany) with a detector type based on Indium Antimonide (InSb). The camera has a spectral range from 1.5–5.1 μm and provides a maximum resolution of 640 × 512 pixels for a frame rate of 100 Hz. Inside the camera a filter wheel is mounted which allows the placement of specific band-pass filters in order to limit the spectral range of the detector. In this case, a 25 mm diameter filter made of sapphire (Al_2_O_3_) with a center wavelength at 3.46 μm and a transmission of 80% supplied by Edmund optics is used. The main application of this filter in the industry is for the detection of CH_4_ and ethanol emissions (hydrogen-carbon bonds), thus being suitable for the application of propane as tracer gas.

Between the IR source and the detector, the quartz column is positioned. One of the main restrictions of the optical technique is the fact that only pseudo-2D columns can be used in order to ensure that all IR emission passes virtually perpendicularly through the column and is not reflected/refracted, which would imply a decrease in the measured intensity. The use of quartz as column material makes it much easier to apply the technique for many different applications, since different sizes and configurations can be easily constructed while a high transmission in the desired wavelength range is always achieved. As a reference case for the development of the technique, a pseudo-2D quartz column with 300, 80 and 8 mm in height, width and depth, respectively, has been built using two quartz plates with a wall thickness of 6 mm. The column, as depicted in Figure 3, is settled in a stainless steel frame which provides the required stability for the measurements. The bottom part of the quartz column is connected to a quartz porous plate distributor of 40 μm pore size and 3 mm thickness. Propane can be either fed through the distributor at the bottom of the column or in a selective way through an injector connected from the top of the column. The injector is a 3 mm stainless steel tube connected to the top of the reactor and ending in a U-shape with the injection point covered by a 100 μm wire mess for a better gas distribution and to avoid particles entering the tube. This column has only been used for the optimization of all the variables involved in the technique.

**Figure 4:**
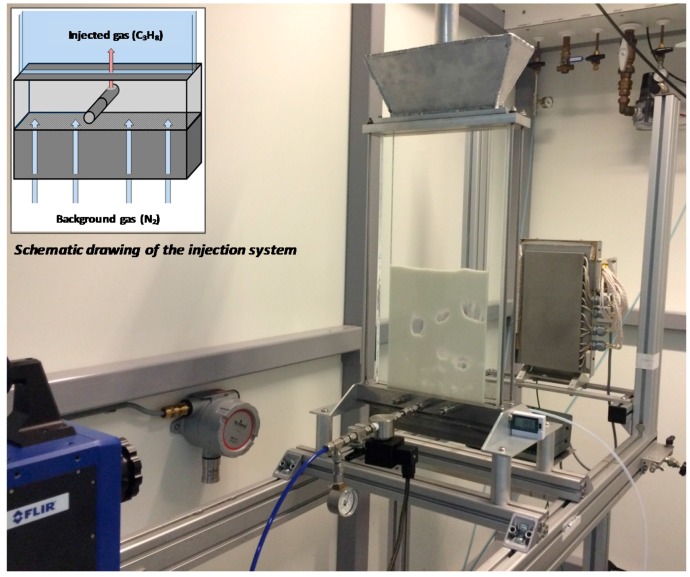
Picture of the scaled-up setup used in this work for mass transfer measurements after optimization of the IR technique and detailed drawing of the injection system for selective feeding of tracer gas.

The suitability of the optimized technique to larger columns has also been proven in this work. A new pseudo 2D quartz column (depicted in Figure 4) with 500, 200 and 4 mm in height, width and depth, respectively, has been built using two quartz plates with a wall thickness of 6 mm. The bottom part of the quartz column is connected to a stainless steel chamber where a stainless steel porous plate distributor with 40 μm pore size and 3 mm thickness has been welded. The distance from the distributor to the bottom part of the quartz column is 25 mm. Just above the porous plate three injectors placed through 6 mm holes have been evenly distributed along the width of the column (depicted in the detail in Figure 4). Through these injectors, propane can be selectively fed inside the reactor. In this case the injectors can work in single-bubble mode by injecting a specific amount of gas or in continuous mode for the study of mass transfer in freely bubbling beds. The depth of the column has been reduced compared to the smaller column in order to reduce the amount of particles present inside the bubbles (because of reduced particle raining). Particles have a very strong IR absorption and would hinder the detection of gas concentrations, thus hampering the measurement of gas concentration profiles in the bubbles, which is important in mass transfer measurements. This bigger quartz column has been used later for mass transfer measurements in fluidized beds.

The technique is based on the use of gas mixtures for the measurement of concentration profiles in the dilute regions of the fluidized bed. In this case, glass beads have been used as dense phase with a mean particle diameter of 0.50 mm, thus showing Geldard B type behavior. The minimum fluidization velocity for these particles has been determined using the standard pressure drop technique obtaining a value of 0.21 m/s at room temperature with N_2_ as fluidizing agent. Actually, N_2_ has been used as secondary gas for the measurement of mass transfer and it has been fed through the porous plate distributor, while propane has been fed through the injection line (except for the calibration where propane-N_2_ mixtures were fed together through the distributor). The amount of gas fed to the column has been controlled via mass flow controllers supplied by Bronkhorst. For the case of single bubble injection, a piston with adjustable volume and pressure is connected in turns to a solenoid valve with an opening time of 0.01 s, which allows fast bubble injection. For mass transfer measurements in fluidized beds the bed is kept at minimum fluidization conditions using N_2_ as background gas and subsequently propane is fed through the injection points either in single-bubble or continuous mode.

### 2.3. Optimization of the IR Transmittance Technique

The calibration of the concentration of the gas tracer is the most important aspect that should be addressed before the technique can be applied for mass concentration measurements. Therefore, the effects of different variables that play an important role on the quality of the calibration have been investigated in detail first, *viz.* steady state operation of the measurement, camera settings and the influence of the distances between IR-column-detector. Also, the reproducibility of the technique has been assessed. This optimization has been carried out with the 80 mm width quartz column reactor depicted in Figure 3.

**Figure 5:**
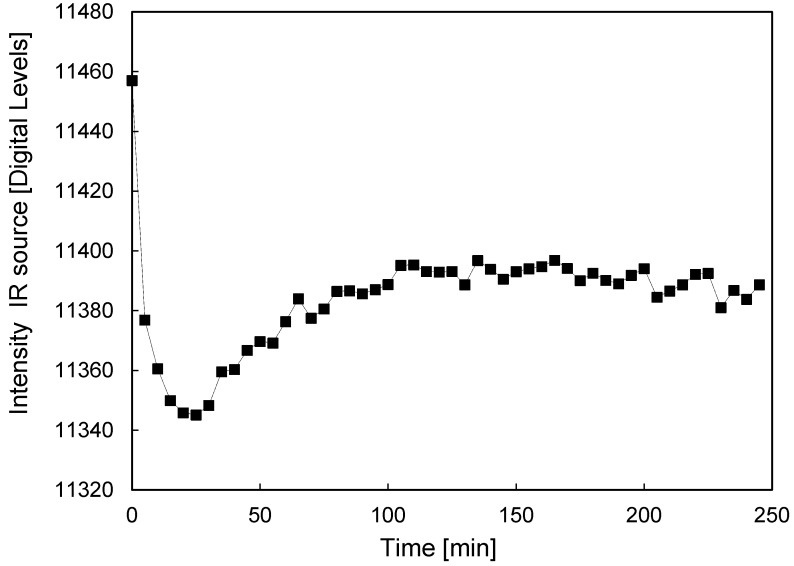
Variation of measured IR intensity as a function of time in an empty quartz column fed with N_2_ once the IR source has reached the target set point (430 °C).

The IR irradiation intensity depends strongly on the temperature of the heated anodized aluminum plate that is used as IR source. Before experiments can be carried out one has to assure that the IR radiation intensity emitted by the heated plate and the intensity detected with the camera has reached steady state conditions. In Figure 5 the IR intensity measured by the camera as a function of time is shown, where the quartz column was placed between the IR source and IR camera while feeding the column with N_2_ (without tracer gas) devoid of particles, after the IR source has reached the target set-point of 430 °C. For this experiment the box where the experimental facility is located has been kept closed in order to avoid any perturbations from outside. As evident from Figure 5, the measured intensity is clearly still changing in time until it reaches steady state conditions after the heated plate has been working at the target set-point for over 2 h. This effect is caused by the continuous heating of the quartz column as a consequence of the heat coming from the IR source. The temperature in the column modifies the measured intensity, and only when a constant temperature, not only of the heated plate, but also of the column, is achieved, steady state operation is obtained. Thus, to achieve reproducible results it is very important to first assure steady state conditions of the IR source and setup.

**Figure 6:**
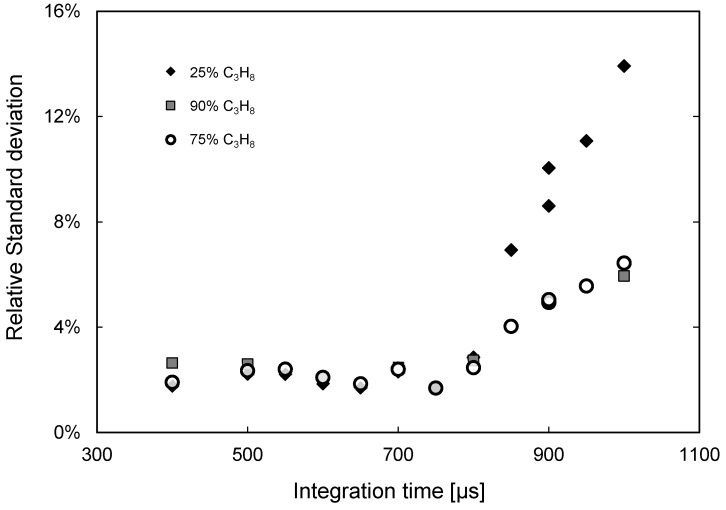
Influence of the camera integration time on the quality of the measurements in relative standard deviation of the measured intensities in the entire image for different C_3_H_8_ concentrations.

Subsequently, the influence of the integration time of the camera is evaluated. This setting is related to the exposure time of the detector to the IR radiation when taking images. In general, the larger the exposure time, the higher the intensity received [32]. However, when the exposure time is too large, the detector might become oversaturated, as already demonstrated by Dang *et al.* [30]. Experiments have been carried out varying the integration time of the camera while feeding the quartz column (devoid of particles) with different N_2_/C_3_H_8_ mixtures (keeping the heated aluminum plate at 430 °C). In this case, first a background image is obtained by feeding pure N_2_ into the column. Subsequently, this background image is subtracted pixel per pixel to the images obtained for the different gas concentrations to correct for inhomogeneous temperature distribution over the IR source and account for the absorbance of the quartz column. In Figure 6, the relative standard deviation in the measured intensity over the entire image is shown as a function of the integration time for different propane concentrations, which clearly shows that the IR camera becomes saturated for an exposure time above 750 μs, irrespective of the flow rate or gas composition used. The oversaturation is related to the sensitivity of the detector, and Figure 6 shows that, in the case of oversaturation, the intensity profile over the reactor also becomes non-uniform, which would increase the error of the technique. Thus, an optimum is found by maximizing the resolution of the technique while avoiding oversaturation (measured as a deviation from a flat profile). For the setup and camera used in this work an integration time of 700 μs has been selected for further experiments.

Finally, the influences of the distances from the camera to the column and the IR source have been evaluated. As previously described, any material at temperatures above 0 K emits IR radiation. Thus the distance of the IR source to the quartz column will affect the temperature of the column, thus changing its transmittance and affecting the measured intensity by the camera. This mainly implies that *I*_0_ is modified. However, for different gas mixtures the relative reduction in the measured intensity (*i.e*., absorbance) will remain the same. Thus, the effect of the distance of the quartz column to the IR source is cancelled out in the normalization procedure, provided that the temperature of the column is constant.

Taking into account the observations and optimizations described above, the calibration of the IR transmittance technique is carried out using different gas mixtures, first using only gas phase and subsequently in gas-solid fluidized beds, to verify the calibration for these systems.

### 2.4. Calibration Method for Tracer Gas Mixtures

**Figure 7:**
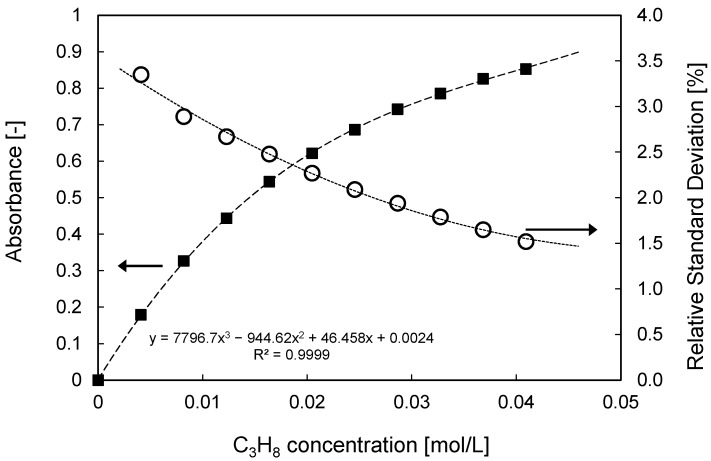
Single gas phase C_3_H_8_ calibration for different gas mixtures using the optimized IR technique and standard deviation over the measurements for different concentrations. Coefficients for the fitting of the calibration are also given.

The calibration measurements have been carried out taking an average background intensity (*I*_0_) of the quartz column fed only with N_2_ of 500 images taken with a frame rate of 50 Hz in order to obtain a representative background image, correcting for small spatial inhomogeneities in the IR emission radiation, and minimizing influences of fluctuations observed in the IR emission and assuring steady state operation (see Figure 5). This background image is used for the determination of the absorbances for different gas mixtures. In total 1000 images at 50 Hz have been taken for any tracer gas mixture composition. As depicted in Figure 7, the propane concentration can be well-correlated using a third order polynomial as a function of the absorbance. The calibration curve shows a linear profile for only very low concentrations, as described by Lambert Beer’s law, whereas it deviates from linearity for higher concentrations attributed to the restriction of movement of the bonds decreasing the extent of absorption. In addition, the absorbance of every single image out of the 1000 images taken for each gas composition has been calculated, with which the standard deviation over all images was determined. This deviation is representative of the maximum standard deviation of the technique and, as also shown in Figure 7, it is quite low. The highest standard deviation is for the lowest tracer gas concentration, but, in any case, lower than about 3%. It should be noted that this error is in the same range as the errors of the mass flow controllers at these low flow rates.

**Figure 8:**
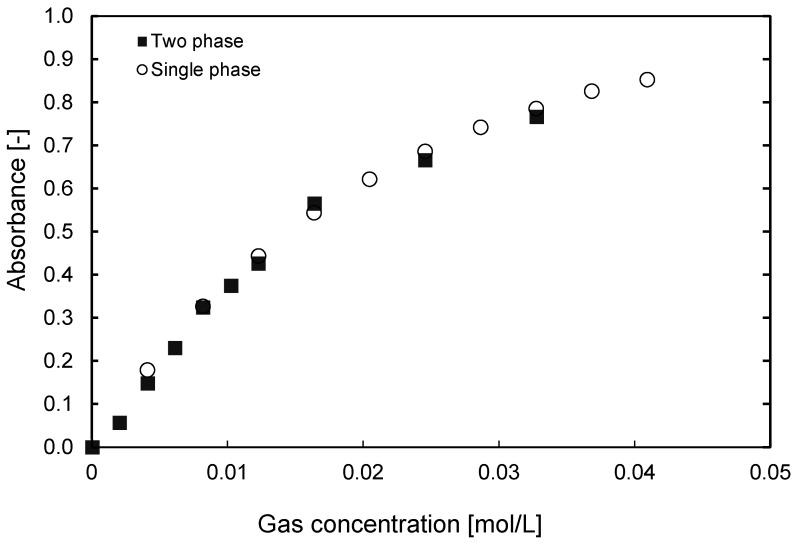
Comparison between the calibration curve of tracer gas concentrations obtained in the empty column and the two phase system using a composed background image.

Subsequently, the calibration was verified for fluidized bed applications. In the top part of Table 2, a distinct discrepancy between the fed and determined concentrations can be observed for the two-phase systems when using the single-phase calibration discussed above, using the empty quartz column fed with N_2_ to determine the background image. The presence of the particles causes a deviation in the intensity measured inside the bubbles, and this deviation does not lie within the error margin of the technique. Therefore, a new calibration method was developed for freely bubbling beds by feeding a known concentration into the fluidized bed and computing the absorbance only for the dilute regions, *i.e*., the gas inside the bubbles, using a sufficiently long recording sequence to cover the entire image of the fluidized bed (typically around 6000 images were needed). The procedure for the determination of the background image is similar, where only N_2_ is fed into the fluidized bed. As can be observed in Figure 8, the thus obtained calibration curve lies on the same curve as the single-phase calibration. What can be concluded from this finding is that the background determination plays the most important role, as the initial intensity (*I*_0_) is changed in the presence of particles. However, the reduction in intensity due to the absorption by the tracer gas is proportional for the two different systems and thus both give the same calibration curves. When the gas concentration is measured using the composed background, the error observed between the concentration fed and measured in the column is less than 10%, indicating a good prediction, especially for higher tracer gas concentrations, as shown in the lower part of Table 2. For the fluidized bed cases, the particles inside the bubbles have been removed, using the same approach as described in detail by Dang *et al*. [30].

As described until now, the determination of the background image represents the most critical part of the experimental technique used in order to obtain good and representative results. First, steady state of the measured IR intensity has to be assured. Subsequently, the composed background including only the dilute regions in the two-phase system is determined, feeding pure N_2_ to the fluidized bed. After that, the calibration (even the one obtained for the single gas phase) can be applied for the measurements. For the experiments in single gas phase (without particles), the background image can be obtained with the empty column fed with N_2_.

In the next section, the developed technique is demonstrated by applying it for preliminary mass transfer studies in membrane separation and fluidized beds. The membrane system is carried out in single gas phase in the presence of gas extraction, aiming at the visualization of concentration polarization. For the fluidized bed case, gas concentrations inside bubbles have been measured to investigate bubble-to-emulsion phase mass transfer phenomena.

## 3. Results and Discussion

### 3.1. Application of the Technique to Visualize Concentration Polarization in Membrane Systems

The use of recently developed high-flux ultra-thin membranes for hydrogen separation in fluidized bed membrane reactors has led to much increased importance in better understanding and describing concentration polarization effects, *i.e*., bed-to-membrane mass transfer limitations [33,34]. When the rate of hydrogen extraction through the membrane is much faster than the transport of hydrogen from the reaction zone to the wall of the membrane, a decrease in the gas concentration occurs near the membrane, thus changing the driving force for gas separation and hence achieving lower permeation rates. This has a clear adverse effect on the system performance. A detailed representation of concentration polarization has thus far not been achieved experimentally, and this is related to the difficulties in measuring gas concentration profiles in the vicinity of the membrane walls. The developed technique allows a whole-field measurement of the concentration with high spatial and temporal resolution, and can thus shine light into concentration polarization phenomena.

For the visualization of concentration polarization in membrane reactors, a porous tube of 6.4 mm in diameter and 70 mm in length with a mean pore size of 40 μm has been used. For this demonstration case, the membrane does not show perm-selectivity for gas separation, but it is used to demonstrate that concentration profiles can be measured next to the membrane wall and that gas separation can, in principle, be visualized. The membrane has a dead-end from one side, while the other side is connected to a mass flow controller for gas extraction, which is connected in turn to a vacuum pump to generate the desired driving force for gas extraction. With this system, it is possible to select the amount of gas extracted via the membrane and mimic actual membrane separation processes at elevated temperatures. The experiment is carried out in single gas phase with selective feeding of C_3_H_8_ through the 3 mm injection point inserted into the column (as depicted in Figure 3) while using N_2_ as background gas.

**Figure 9:**
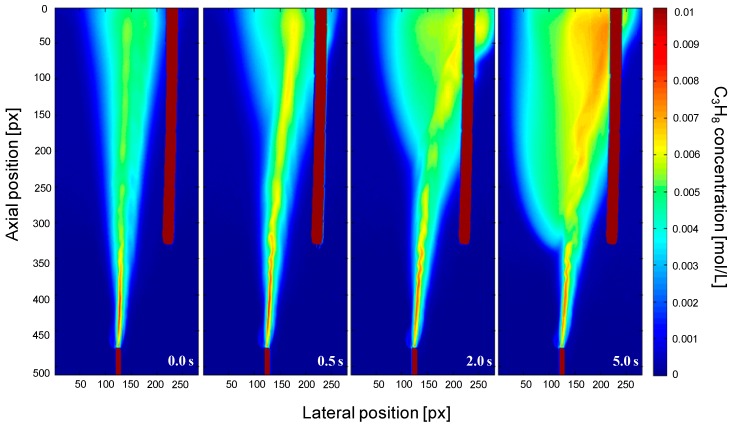
Tracer gas concentration profiles for gas extraction through a porous membrane connected to a vacuum pump using the IR-technique at different moments in time after turning on the vacuum pump.

In Figure 9 a case of 10% gas extraction through the porous membrane is presented at different moments in time after the vacuum at the permeate side has been imposed. In the left part of the figure, the gas extraction system was not connected and thus the C_3_H_8_ rises through the bed showing dispersion of the jet, as usual in the laminar flow regime. Once the vacuum pump is connected, a driving force for gas extraction via the membrane is created, causing bending of the flow towards the membrane. This effect becomes even more pronounced for longer times after turning on the vacuum pump. Most of the gas that is extracted through the membrane is coming from the N_2_ background gas, thus increasing the C_3_H_8_ concentration, as can be clearly observed in the most right-hand figure. However, it is important to note that the observed increase in concentration was not caused by differences in perm-selectivity of the membrane for C_3_H_8_ and N_2_; however, this test experiment has indicated that the developed technique may be used to visualize and quantify concentration polarization effects in membrane separators and reactors.

### 3.2. Scaling-Up of the Experimental Setup for Freely Bubbling Beds

One of the main advantages of the novel IR technique is the use of an inexpensive system (except for the high-speed IR camera), thus suitable for being applied to larger columns that better represent actual commercial or industrial systems, avoiding wall effects. In this section, the use of the developed IR technique is described to investigate mass transfer phenomena in a larger fluidized bed using the bigger quartz column (described in Section 2.2).

Studies on the mass exchange between the bubble and emulsion phases in freely bubbling gas-solid fluidized beds have been performed in the literature mainly using either a decomposition reaction or using the injection of a tracer gas (stimulus-response measurements). When using a decomposition reaction, the obtained conversion was compared to the conversion obtained with fixed-bed reactors. While these experiments give satisfactory results for the investigated experimental set-up, it is very difficult or even impossible to directly relate the experimental results to the prevailing flow phenomena and extrapolate the results to different systems and/or different operating conditions. The tracer gas experiments allow for a more detailed study of the flow behaviour of the gas exchange between bubble and emulsion phases. However, most tracer gas studies used internal probes and obstructions to analyse gas concentrations. These internals or extraction points influence the results and assume an ideally mixed phase. The mass transfer in most phenomenological models is described by the volumetric mass transfer coefficient *K_be_*, for which a semi-empirical correlation as described in Equation (4) was developed [2].

(4) $$K_{be}=\frac{4}{d_{b}}\left(0.6D_{C_{3}H_{8}}^{0.5}{\left(\frac{g}{d_{b}}\right)}^{0.25}+\frac{2u_{mf}}{\pi }\right)$$

In the works of Patil *et al*. and Dang *et al*. [29,30], it is shown that the bubble diameter does not remain constant and a non-uniform concentration profile inside the bubble was measured. These are two assumptions often used in phenomenological models for the prediction of the bubble-to-emulsion phase mass transfer coefficient. Therefore, a more detailed investigation of these two issues is of importance to develop a better description of the mass transfer phenomena in bubbling fluidized beds.

**Figure 10:**
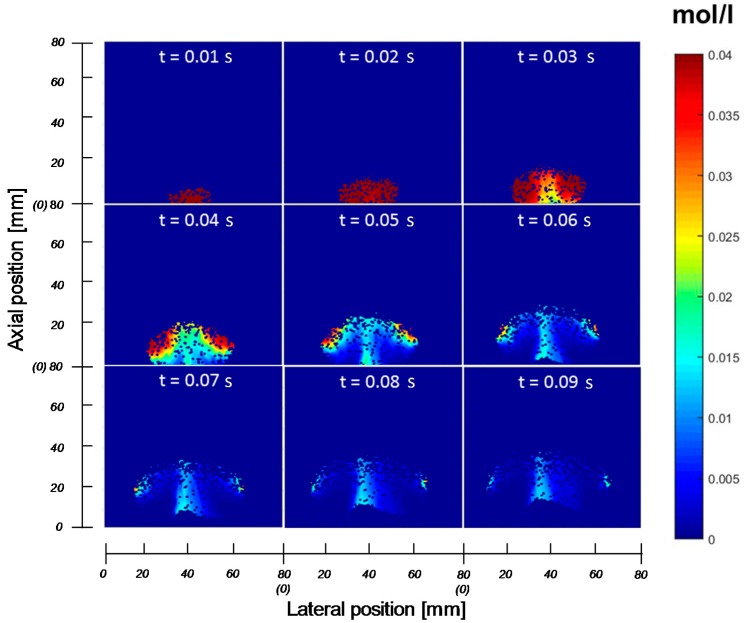
Tracer gas concentration profiles inside an injected bubble in a bed at minimum fluidization conditions using the IR technique as a function of time (t) in seconds.

The novel developed technique is used in this section for the study of the bubble-to-emulsion phase mass exchange coefficient for single injected bubbles. The tracer gas is injected in controlled volumes via a single orifice above the distributor plate, while the bed is initially at incipient fluidization conditions in absence of tracer gas. The injected tracer gas will form a bubble containing the tracer gas and snapshots of the tracer concentration profiles inside the bubble at different moments in time after bubble detachment can be analysed, where the variation of the total tracer gas concentration in the bubble in time is used to determine the mass transfer coefficient. In Figure 10 the generation and propagation of an injected bubble into a fluidized bed at incipient fluidization conditions consisting of glass beads with a particle size ranging from 0.62 to 0.72 mm at different moments in time is shown.

**Figure 11:**
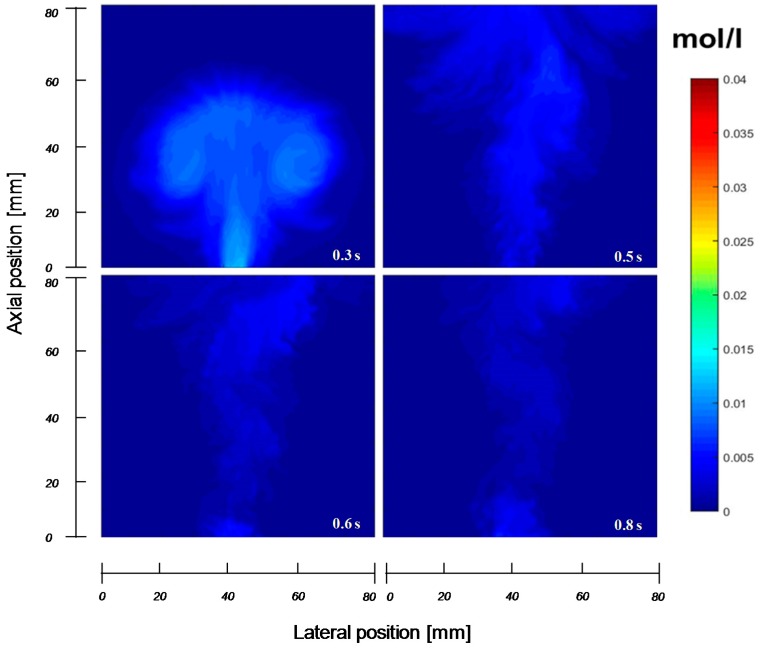
Tracer gas concentration profiles from gas injection into an empty column fed with N_2_ as a function of time.

In the first three frames the bubble is formed with the tracer gas coming from the orifice. After 0.03 s, nitrogen flows into the bubble from the bottom part of the bubble as convective gas flow. In the next frames most of the tracer gas is flushed out of the bubble, except for the regions on the left and right top part of the bubble, where there are still some high concentration regions present, mainly because of some diffusion limitations. Further, there is through flow of tracer gas in the middle of the bubble, although it is not clear whether this is caused by backflow of gas via the vortices extending in the emulsion phase on the sides of the bubble or due to some remaining flaring from the injection point. Unfortunately, the gas concentration profiles in the emulsion phase cannot be measured with the developed IR technique. However, from separate experiments with tracer gas injection into an empty column fed with nitrogen, it can be shown that the injection point indeed flares after tracer gas injection. A small sequence of concentration profiles during an injection into a column without particles is shown in Figure 11. The amount of nitrogen fed into the column was the same as the amount to achieve incipient fluidization for the results shown in Figure 10. It can be seen that the gas injection provokes a “blob” of gas, which subsequently moves up rapidly out of the image, implying that the presence of particles clearly influences the gas velocity. Furthermore, it is observed that the gas tracer concentration just after the gas injection in case of the empty column is much lower, indicating that the presence of the particles also influences the extent of gas mixing. After the tracer gas injection, there is still gas leaking from the injection point, which might explain the effect observed in the bubble in Figure 10. Although this effect should be carefully considered, the amount of gas flaring from the injector is insignificant for the determination of mass exchange coefficient in this experiment.

The volumetric mass transfer coefficient has been determined for the recordings depicted in Figure 10 employing the same procedure as described previously by Dang *et al*. [30], where the molar balance of the amount of tracer gas in the bubble is solved in time. The experimental mass transfer coefficient obtained for this case is shown in Table 3.

Experimental results have been compared to the theoretical mass exchange coefficient given in Equation (4) using the bubble properties determined from the experiment. The results show that the new technique can be used for evaluation of the mass transfer in fluidized bed reactors. The deviation between the measured values and the theoretical one is related mainly to two factors: on the one hand the experiment allows to measure concentrations with higher spatial resolution, which in itself allows better prediction of the mass transfer coefficient; on the other hand, the experiment relates to the case of single bubble injection which is different from the freely bubbling case considered in the theoretical one. In a future work we will extend the technique to freely bubbling beds.

## 4. Conclusions/Outlook

This work has been focused on the further development and optimization of an IR-technique for concentration measurements in larger scale gas-phase or gas-solid fluidized systems. In the first part, the selection of the most appropriate system has been widely discussed and the effects of the most important parameters have been carefully investigated. A standard procedure has been developed for proper calibration to ensure reproducibility of the results, which include the determination of the composed background for a two-phase system, after assuring steady state operation of the IR intensity measured (constant temperature of the quartz reactor). This procedure takes in total about 3–4 h, and it always has to be done prior to the measurements. However, the actual concentration measurements are very fast. Despite the fact that the developed technique can only be applied to pseudo-2D columns and there is limited freedom in the selection of the reactor material and tracer gas, the technique has some clear advantages over other experimental techniques: in particular, it is a non-invasive monitoring technique with a high temporal and spatial resolution applicable to many different applications.

In the second part, the developed technique has been applied to two interesting transport phenomena in the chemical engineering field. Concentration polarization and effects of gas extraction have been visualized for the first time, showing great potential of the technique to be applied to a more detailed study in the field of membrane separators and reactors. Furthermore, the use of a bigger quartz column has demonstrated that the technique can also be applied to study mass transfer phenomena in larger-scale fluidized beds with decreased wall effects.

The novel IR-technique has shown great potential for better understanding of the fundamentals of fluidized bed reactors and membrane reactors. In a future work a detailed investigation on the mass transfer in freely bubbling fluidized bed reactors is planned, and a fundamental study on concentration polarization effects in membrane reactors using a perm-selective membrane for gas separation at low temperatures is envisaged. In addition, the combination of mass transfer and hydrodynamics is also in progress by triggering two different cameras (one VIS and one IR camera) combining the developed technique to Particle Image Velocimetry (PIV) coupled with Digital Image Analysis (DIA).

## Figures and Tables

**Table 1:** Different phenomenological models described in the literature.

Level	Description	References
I	Parameters are constant along the bed. Parameters are not related to the bubble behaviour	[16]
II	Parameters are constant along the bed. Parameters are related to the bubble size, which is adjustable	[17]
III	Parameters are related to the bubble size. Bubble size is varied along the bed height	[18]

**Table 2:** Measurement of the error for two phase system with different backgrounds.

**Empty Column Background**			
C_3_H_8_ (%)	Input	measured	Absolute deviation
	(mol/L)		
10	0.004092	0.0106	158.3%
21	0.00859	0.0157	85.0%
32	0.013169	0.0224	70.1%
**Composed Background**			
C_3_H_8_ (%)	Input	measured	Absolute deviation
	(mol/L)		
6	0.00246	0.00279	13.5%
11	0.00450	0.00490	8.85%
16	0.00655	0.00717	9.51%
21	0.00859	0.00891	3.62%
28	0.01146	0.01213	5.90%

**Table 3:** Experimental bubble-to-emulsion mass transfer coefficients.

Injected volume (mL)	Velocity (m/s)	K_be_ (1/s)	R	Average d_b_ (mm)	Theoretical K_be_ (1/s)
3.96	16.4	34.2	0.99	37.4	23.9
